# An in vivo accelerated developmental myelination model for testing promyelinating therapeutics

**DOI:** 10.1186/s12868-022-00714-y

**Published:** 2022-05-25

**Authors:** Karen Lariosa-Willingham, Dmitri Leonoudakis, Timo Bragge, Laura Tolppanen, Antti Nurmi, Megan Flanagan, Janelle Gibson, David Wilson, Jennifer Stratton, Kimmo K. Lehtimäki, Diana Miszczuk

**Affiliations:** 1 Teva Pharmaceutical Industries Ltd, Redwood City, CA 94063 USA; 2 Charles River Discovery Services, Neulaniementie 4, 70210 Kuopio, Finland; 3 Sword Diagnostics, Chicago, IL 60612 USA

**Keywords:** Promyelinating, Diffusion tensor imaging, Fractional anisotropy, Thyroxine, Rat, MRI, Resonance Raman spectroscopy, ELISA

## Abstract

**Background:**

Therapeutic agents stimulating the process of myelination could be beneficial for the treatment of demyelinating diseases, such as multiple sclerosis. The efficient translation of compounds promoting myelination in vitro to efficacy in vivo is inherently time-consuming and expensive. Thyroid hormones accelerate the differentiation and maturation of oligodendrocytes, thereby promoting myelination. Systemic administration of the thyroid hormone thyroxine (T4) accelerates brain maturation, including myelination, during early postnatal development. The objective of this study was to validate an animal model for rapid testing of promyelinating therapeutic candidates for their effects on early postnatal development by using T4 as a reference compound.

**Methods:**

Daily subcutaneous injections of T4 were given to Sprague Dawley rat pups from postnatal day (PND) 2 to PND10. Changes in white matter were determined at PND10 using diffusion tensor magnetic resonance imaging (DTI). Temporal changes in myelination from PND3 to PND11 were also assessed by quantifying myelin basic protein (MBP) expression levels in the brain using the resonance Raman spectroscopy/enzyme-linked immunosorbent assay (RRS-ELISA) and quantitative immunohistochemistry.

**Results:**

DTI of white matter tracts showed significantly higher fractional anisotropy in the internal capsule of T4-treated rat pups. The distribution of total FA values in the forebrain was significantly shifted towards higher values in the T4-treated group, suggesting increased myelination. In vivo imaging data were supported by in vitro observations, as T4 administration significantly potentiated the developmental increase in MBP levels in brain lysates starting from PND8. MBP levels in the brain of animals that received treatment for 9 days correlated with the FA metric determined in the same pups in vivo a day earlier. Furthermore, accelerated developmental myelination following T4 administration was confirmed by immunohistochemical staining for MBP in coronal brain sections of treated rat pups.

**Conclusions:**

T4-treated rat pups had increased MBP expression levels and higher MRI fractional anisotropy values, both indications of accelerated myelination. This simple developmental myelination model affords a rapid test of promyelinating activity in vivo within several days, which could facilitate in vivo prescreening of candidate therapeutic compounds for developmental hypomyelinating diseases. Further research will be necessary to assess the utility of this platform for screening promyelination compounds in more complex demyelination disease models, such us multiple sclerosis.

**Supplementary information:**

The online version contains supplementary material available at 10.1186/s12868-022-00714-y.

## Background

Many central nervous system disorders stem from pathological changes in myelin structure, which can be broadly categorized into dysmyelination, when myelin is malformed and defective, and demyelination, when the initially normal myelin becomes destroyed [1]. Dysmyelinating conditions, also known as leukodystrophies, usually have a strong genetic component and, therefore, exhibit early developmental signs [2–4]. Myelin dysfunction in dysmyelinating conditions may be caused by a lack of a particular myelin constituent, e.g., of proteolipid protein 1 in Pelizaeus–Merzbacher disease [5], delay in myelination, as in Allan-Herndon-Dudley syndrome featuring mutations in the *SLC16A2* gene that encodes a thyroid hormone transporter [6], or metabolic errors, as in Canavan disease, which is caused by mutations in the *ASPA* gene encoding aspartoacylase, an enzyme enriched in oligodendrocytes [7]. Demyelinating disorders, such as multiple sclerosis (MS), neuromyelitis optica and acute disseminated encephalomyelitis, usually occur in adults and may be caused by autoimmune processes and infections, with a contribution of genetic, environmental, and dietary factors [1, 8–13]. Furthermore, other conditions, such as genetic leukoencephalopathies and certain metabolic disorders, also present with myelination defects, though myelin disturbances follow abnormal neuronal development, neuronal loss and profound systemic abnormalities [14]. Disorders such as autism, schizophrenia, and Williams-Beuren syndrome have recently been associated with hypomyelination, revealing new roles of oligodendrocytes and myelin in the development of the nervous system [15–17]. Moreover, behavioral and myelin deficits in a mouse model of Williams-Beuren syndrome were shown to be rescued by the remyelination agent clemastine [15]. Irrespective of the etiology, diseases associated with the dysregulation of myelination processes generally lack adequate treatments. The need for novel therapeutics is critical because myelin disorders are often disabling and life-threatening for patients, and also come with an extremely high societal burden.

Previously, we and others have published methods for multi-well plate-based primary in vitro assays for high throughput screening of promyelinating compounds [18–26]. Clemastine was identified as a potential remyelinating agent in such a predictive screening assay [27], and it has since showed clinical efficacy in patients with optic neuritis and MS [28, 29]. In addition to in vitro assays, it would be extremely helpful to establish rapid in vivo tests, predicting the promyelinating efficacy of promising compound candidates in a matter of days, prior to testing in long and costly in-life models of myelination disorders for weeks and months.

The thyroid hormones triiodothyronine and its precursor thyroxine (T4) are necessary for the normal development of myelin-generating oligodendrocytes [30, 31]. The stimulatory role of T4 on myelination is known since the 1960s [32]. Supplementation with thyroid hormones in several demyelination models restored expression of the myelin basic protein (MBP) [33], promoted maturation of oligodendrocyte precursor cells [34, 35], regulate the timing of oligodendrocyte differentiation and development [36] and accelerated remyelination [37–39]. However, chronic exposure to T4 eventually causes oligodendrocyte death by apoptosis, which precludes therapeutic use of this hormone [40]. In the present study, to avoid adverse effects of the chronic T4 exposure, we determined changes in the myelination status in neonatal rats after subcutaneous injections of T4 for a limited period of 9 days to assess the suitability of this neonatal accelerated developmental myelination model for rapid screening of promyelinating drugs.

## Materials and methods

### Animals

 All animal experiments were performed as specified in the license authorized by the National Animal Experiment Board of Finland and according to the National Institutes of Health (Bethesda, MD, USA) Guidelines for the Care and Use of Laboratory Animals. A total of 108 Sprague Dawley rat pups (CD^®^ Sprague Dawley dams were obtained from Charles River Laboratories, Sulzfeld, Germany) of both sexes were used in the study. Pregnant dams and pups were housed in open top cages in a light-controlled environment (lights on at 07:00 am and off at 8:00 pm) at a temperature of 22 ± 1 °C and relative humidity of 40–70%. All dams were monitored for labor and delivery. All animals had access to chow (Teklad Global 2016, Envigo) and water *ad libitum*.

All pups used in the in vivo diffusion tensor MRI measurements were anesthetized with isoflurane to perform the in life measurements. Euthanasia was performed by decapitation on all pups as an end of live procedure the next day following the last injection of T4 or vehicle.

### Developmental model of hyperthyroidism-enhanced myelination

**Fig. 1:**
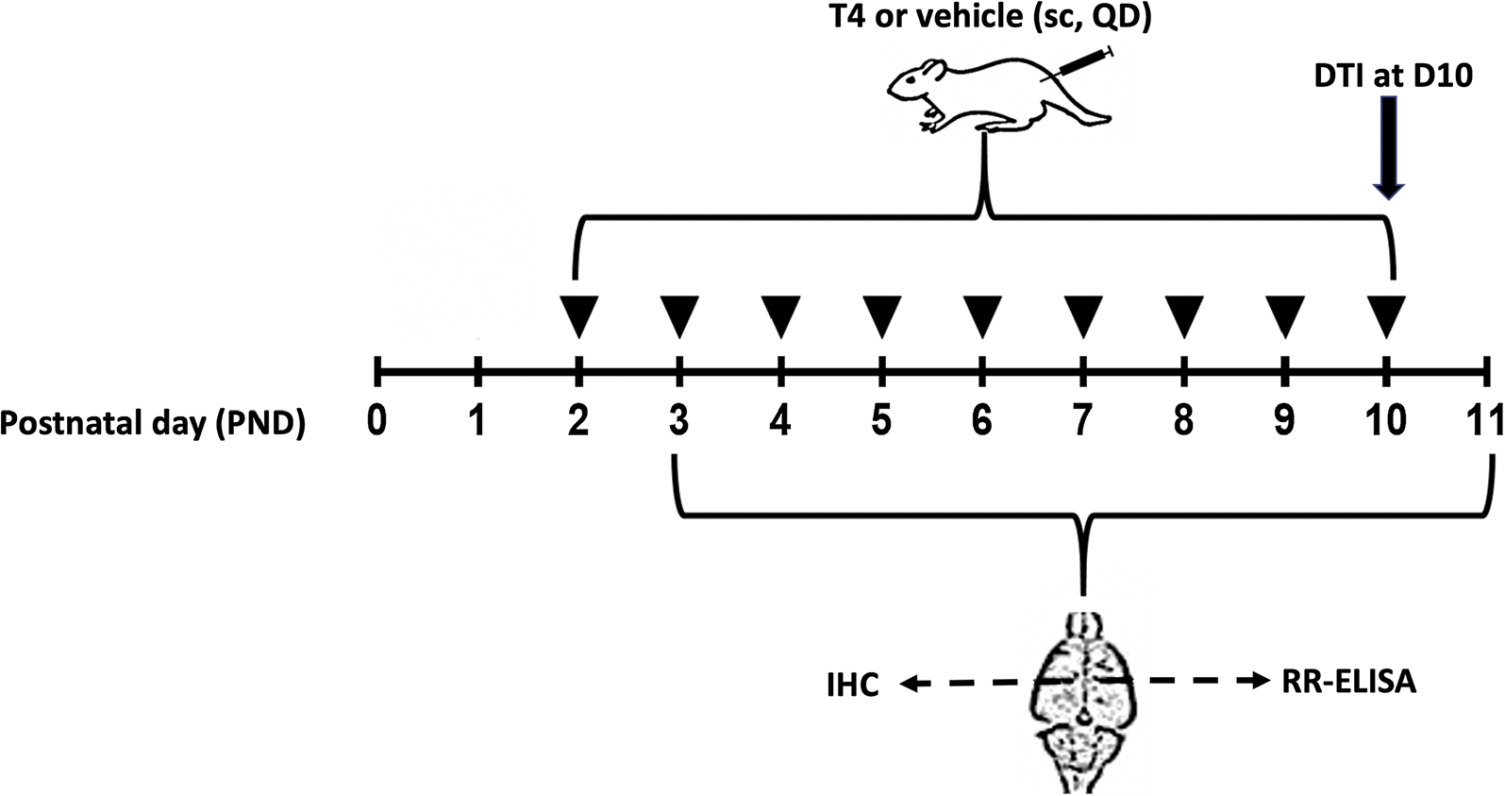
Study design. A total of 68 rat pups were divided into 18 experimental groups of 5–7 pups/group, which received from one to nine daily injections of thyroxine (T4) or vehicle, starting from postnatal day 2 (PND2). Groups 1 and 2 received T4 or vehicle, respectively, on PND2 and were sacrificed on PND3, groups 3 and 4 received T4 or vehicle, respectively, on PND2 and PND3 and were sacrificed on PND4, etc. Animals were sampled daily from PND3 to PND11, and brain hemispheres were collected for IHC and RR-ELISA experiments. Diffusion tensor MRI (DTI) was performed on PND10 for vehicle and T4 rat pups (group 17 and group 18)

Given that standardized T4 dosing regimen to achieve hyperthyroidism has not been firmly established [41, 42], we performed pilot experiments with early postnatal rat pups, testing effects of T4 at doses of 0.1 and 0.3 mg/kg, and found that the former dose was able to significantly increase myelin content. Because T4 regulates oligodendrocyte development timing, and because repeated T4 injections into newborn rats had been shown to induce appearance of oligodendrocytes in structures such as the developing optic nerve [36], we adopted the same strategy for our model. Therefore, developmental hyperthyroidism-enhanced myelination was induced in CD rat pups by daily subcutaneous injections over the hip area of L-thyroxine sodium salt pentahydrate (T4; 0.1 mg/kg; Sigma-Aldrich, St Louis, MO, USA). The study design is presented in Fig. 1. Briefly, rat pups were divided into 18 experimental groups of 5–7 pups/group, which received from one to nine daily injections of T4 or vehicle, starting from postnatal day 2 (PND2). A 10 µL Hamilton syringe with a 30-gauge ½” needle was used to subcutaneously inject dissolved T4 or vehicle (water for injections, Braun, Melsungen, Germany) at 0.5 mL/kg body weight into the hip area of rat pups. During weighting and injections, pups were kept on a homeothermic blanket (62 W Thermo Mat, Lucky Reptile, Waldkirch, Germany) at 37 °C, and the site of injection was alternated daily. After every injection, pups were immediately returned to their dams. Next day after the last injection of T4 or vehicle, the pups from groups 1–18 were decapitated at PND3–PND11, respectively (groups 1 and 2 — on PND3, groups 3 and 4 — on PND4, etc., Fig. 1). Animals were observed twice a day, at 7–10 a.m. and 3–6 p.m., during the course of the study to ensure adequate maternal care and survival of the pups during the follow-up period. Body weight and development of teeth were recorded daily during T4 or vehicle dosing procedure.

### White matter assessment by diffusion tensor magnetic resonance imaging

Diffusion tensor magnetic resonance imaging **(**DTI) acquisitions were performed in animals from groups 17 and 18 at PND10, after they had received eight daily injections, but before their last (ninth) injection, using a horizontal 11.7 T magnet with a bore size of 160 mm, equipped with a gradient set capable of maximum gradient strength of 750 mT/m and interfaced to a Bruker Avance III console (Bruker Biospin GmbH, Ettlingen, Germany). A volume coil (Bruker Biospin GmbH, Ettlingen, Germany) was used for transmission, and a surface phased array coil (Rapid Biomedical GmbH, Rimpar, Germany) was used for receiving. The pups were anesthetized using isoflurane (5% for induction, 1.5% for maintenance in 300 mL/min N_2_/O_2_), fixed to a custom-built head holder, and positioned in the magnet bore in a standard orientation relative to gradient coils. Animal temperature was kept at 36–37 °C with a warm water circulation heating blanket. After acquisition of fast localizer images, DTI was performed using a 4-segment echo-planar imaging sequence with 30 diffusion directions (b-values 0 and 970 s/mm^2^), time-to-repeat of 4000 ms and echo time of 23.5 ms. A field-of-view of 12.80 × 10.24 mm^2^ was used with a matrix of 160 × 128, resulting in the 80 μm in-plane resolution. Fifteen 0.6 mm slices were acquired with six averages. Following eddy-current correction and brain masking, the diffusion tensor was calculated using dtifit program within FSL software (https://fsl.fmrib.ox.ac.uk/fsl/fslwiki/) on the basis of the default linear regression model. Outputs of the dtifit program, fractional anisotropy (FA) and mean diffusivity (MD) were used as such. The 1st eigenvalue (L1) represented axial diffusivity (AD), whereas 2nd and 3rd eigenvalues (L2 and L3, respectively) were averaged to provide radial diffusivity (RD) values. Diffusion indices for white matter (WM) changes were analyzed bilaterally for the anterior part of the anterior commissure, corpus callosum (forceps minor, forceps major, genu, body and splenium), external capsule, internal capsule, optic tract, and cerebellar peduncle. To account for more global and subtle changes in FA, a single large region of interest (ROI) encompassing all forebrain WM structures and grey matter was manually delineated for each DTI-scanned subject (example ROI shown in Additional file 2). Individual histogram distributions of FA from that ROI were produced in 0.01 bin widths between 0 and 1, normalized (sum of all bin contributions equals 1) and group average histogram distributions produced. Having observed that the average value of T4 group distribution had an apparent shift relative to the vehicle group value, we fitted histogram distributions of each animal using three-parameter Burr distribution [43], yielding fit parameters (*c, k, α*) that allowed quantitative description of the histogram shift in terms of the mean, median, and mode values. Individual histogram distributions for all subjects, Burr-distribution fits and fitting parameters are shown for T4 and vehicle groups in Additional files 3 and 4, respectively.

### Endpoint sampling

Pup brains were dissected at PND3–PND11 and weighed. After weighing, the right hemisphere was snap-frozen in liquid nitrogen and stored at − 80 °C until processing for the preparation of brain lysates for MBP analysis by using resonance Raman spectroscopy ELISA (RRS-ELISA). The left hemisphere was immersion-fixed in 4% paraformaldehyde in 0.1 M phosphate buffer for 18–24 h followed by cryoprotection in 30% sucrose in phosphate buffer for 2–3 days. Cryoprotected hemispheres were frozen in liquid nitrogen and stored at − 80 °C until quantitative immunohistochemical (IHC) analysis.

### MBP measurement by RRS-ELISA

Whole brain lysates were prepared by lysing homogenized brain tissue in RIPA lysis buffer (Cell Signaling, Danvers, MA, USA) supplemented with Halt™ protease and phosphatase inhibitors (ThermoFisher Scientific, Waltham, MA, USA). Homogenization was performed using Tissue Lyser II (Qiagen, Hilden, Germany) for 2 min at 25 Hz. The homogenates were centrifuged for 5 min at 5000×*g* at 4 °C. The supernatants were stored frozen at − 80 °C. MBP quantification was performed using an ELISA enhanced resonance Raman platform (Product No. TEVSK-MBP00-01, Sword Diagnostics, Chicago, IL). Plates were washed with a microplate washer (Biotek 405 LS). Fluorescent signal was detected on a microplate reader (BioTek Cytation 5) with an optimal Red-shifted PMT (filter mode excitation 530/24 nm, emission 730/40 nm). Softmax Pro Version 6.5.1 (Molecular Devices, San Jose, CA, USA) was used for data analysis.

### MBP detection by quantitative immunohistochemistry

Brain hemispheres from rat pups sacrificed at PND3–11 were embedded, sectioned, stained, and imaged using MultiBrain^®^ Technology (Neuroscience Associates, Knoxville, TN), during which multiple brain samples are embedded within gelatin blocks providing uniform exposure of brain tissue to sectioning and staining conditions. Cryo-sectioning was performed at 40 μm in the coronal plane through the full-length of mouse brain hemispheres. Sections were stained with a primary anti-MBP mouse antibody (clone SMI-99, BioLegend, San Diego, CA), incubated a biotinylated secondary anti-mouse horseradish peroxidase-conjugated antibody with nickel (Vector Laboratories, Burlingame, CA), counterstained with neutral red and mounted on gelatinized glass slides. Slices from pups sacrificed at PND5 and PND7–11 were utilized for image quantification, whereas slices from pups sacrificed at PND3, PND4, and PND6 groups were not analyzed due to negligible MBP expression. Each slide was digitally imaged at 10× magnification and quantified using histopathology image analysis software platform Halo™ (Indica Labs, Tacoma Park, MD, USA). Briefly, we identified three adjacent sagittal cross-sections of approximately the same region of each brain hemisphere from at least two animals per group and used data from them for analysis. The entire cross-sectional region in each image was defined as the ROI. Minor adjustments were applied from the initial image analysis algorithm (Area Quantification v1.0), allowing increased pixel sensitivity by using a low threshold setting. After absolute value background subtraction and thresholding, all pixels of the regions highly positive for MBP were identified and segmented from unstained tissue regions using a tissue classifier module. Immunostaining data were expressed as the percentage of the whole hemisphere area occupied by MBP-positive tissue.

### Data analysis

Statistical data analysis was performed using Prism 9 software (GraphPad Software, Inc., La Jolla, CA, USA). The fractions of pups with teeth eruption at PND9 were compared using the Fisher’s exact test. MBP levels determined by RRS-ELISA were analyzed using two-way analysis of variance (ANOVA) with treatment and age as factors, followed by the *post hoc* the Tukey´s multiple comparison test. Results of IHC assessment of MBP were analyzed using using the Student’s *t*-test and the correction for multiple comparisons was done by controlling for the false discovery rate (FDR; set at q = 0.1) by using the Benjamini–Krieger–Yekutieli procedure. Differences were considered statistically significant if adjusted *P* < 0.05.

In DTI analysis, FA, RD, AD and MD values in each of the 10 brain areas were compared using the Student’s *t*-test and the correction for multiple comparisons was done by controlling for the false discovery rate (FDR; set at q = 0.1) by using the Benjamini–Krieger–Yekutieli procedure implemented in Prism 9. Global FA histogram distribution shift was described as individual subject’s medians from Burr-distribution fits and group comparisons were statistically tested using the Welch’s *t*-test. Data are presented as the mean (M) ± standard error of the mean (SEM) or standard deviation (SD). Differences were considered statistically significant if adjusted *P* < 0.05.

## Results

### Developmental changes

Tooth eruption is a timed developmental process in which teeth move from the bony crypt to reach their functional position in the oral cavity [44]. Thyroid hormones, including T4, have been reported to affect the rate of tooth eruption in rodents [45] and humans [46]. In the current study, as expected, T4 accelerated teeth eruption rate. In particular, out of the 11 T4-treated rat pups that had not been dissected by PND9, 9 pups had tooth eruption, whereas among the 11 vehicle-treated pups, there was only one pup with erupted teeth (*P* = 0.0019, Fisher’s exact test). Body weight increased with age at similar rates in vehicle- and T4-treated pups (data not shown), and no animal welfare issues were observed in the study.

### DTI of cerebral WM

**Fig. 2:**
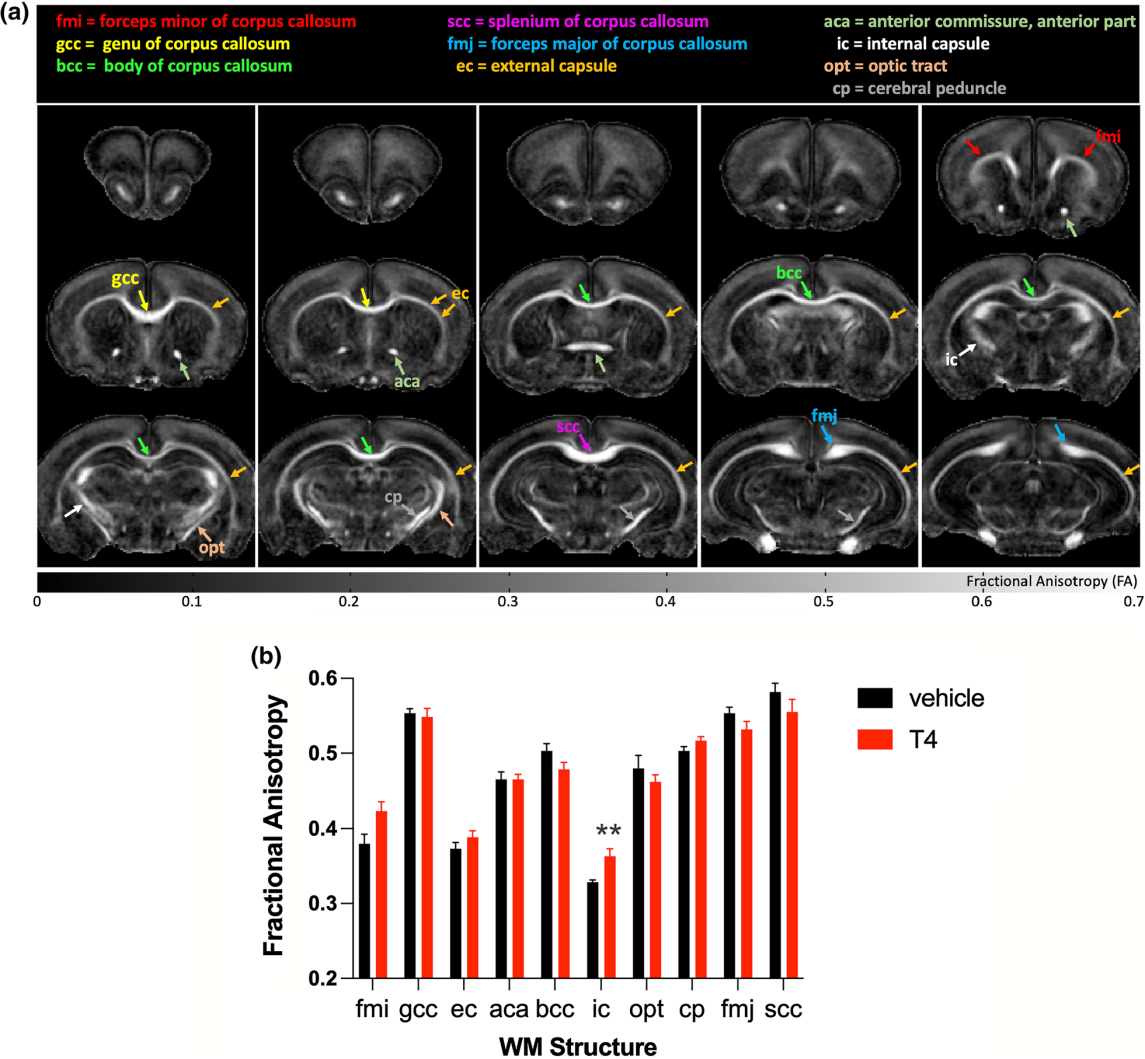
Diffusion tensor MRI on major white matter structures. **a** Fractional anisotropy (FA) map with highlighted 10 major white matter brain regions obtained by diffusion tensor imaging in a representative thyroxine (T4)-treated rat at PND10. Scaling of the FA map is between 0 and 0.7. **b** FA values in 10 different areas of the white matter in rat pups at PND10 following nine daily administrations of T4 (0.1 mg/kg, *n* = 6) or vehicle (water, *n* = 6). Data are presented as the mean ± SEM. Statistical significance is indicated as follows: **P < 0.01 (passed correction for the false discovery rate at q set at 0.1). *fmi* forceps minor of corpus callosum, *gcc* genu of corpus callosum, *bcc* body of corpus callosum, *scc* splenium of corpus callosum, *fmj* forceps major of corpus callosum, *ec* external capsule, *aca* anterior part of anterior commissure, *ic* internal capsule, *opt* optic tract, *cp*. cerebellar peduncle

**Fig. 3:**
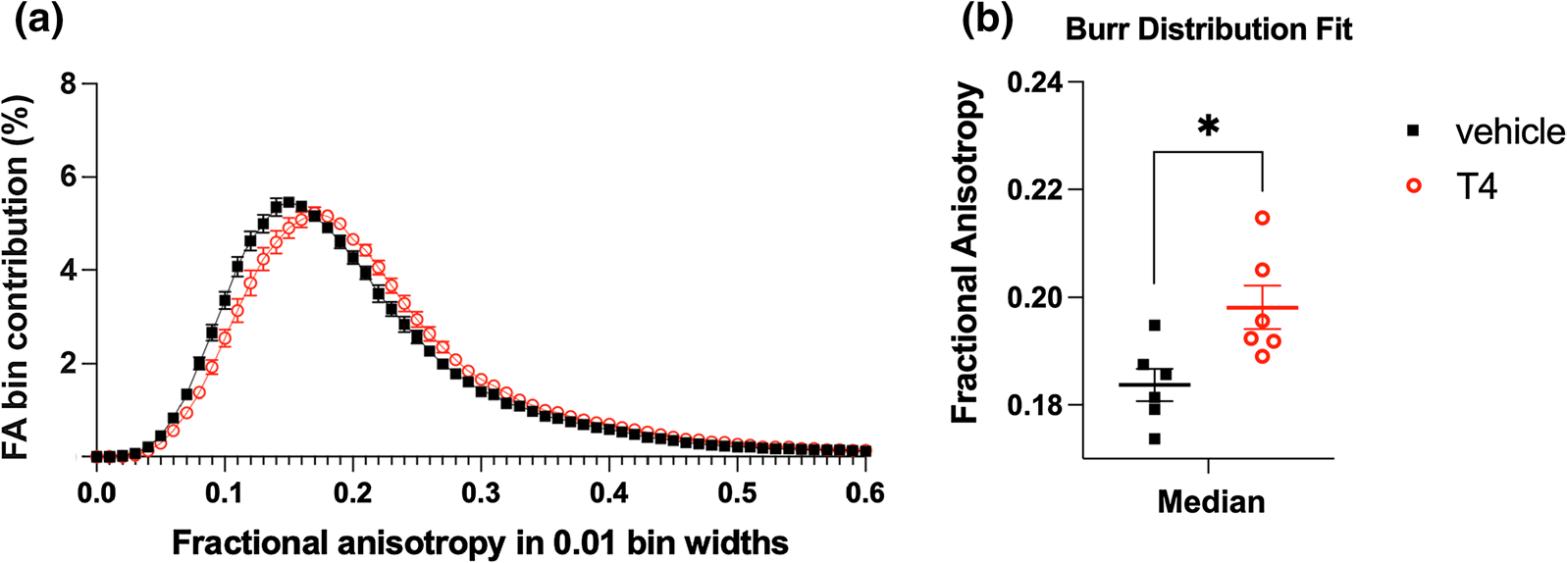
Global distribution of fractional anisotropy (FA) values in the brains of rat pups at PND10. **a** Group averages of normalized FA histograms for thyroxine (T4) and vehicle rat pups at PND10; distribution of T4 group FA values shows an apparent shift to higher values. **b** Analysis of the FA shift by the comparison of Burr distribution fit medians for individual subjects. Data are presented as the mean (thick solid line) ± SEM. *P < 0.05 (Welch’s *t*-test). The T4 group (n = 6) received T4 at 0.1 mg/kg and the vehicle group (n = 6) received water

A representative FA map of a rat pup at PND10 is shown in Fig. 2a with annotations for the major structures used in manual ROI analysis. We found that FA was increased in the forceps minor of corpus callosum (*t*_10_ = 2.54, unadjusted *P* = 0.0294) and internal capsule (*t*_10_ = 3.38, unadjusted *P* = 0.007) of rat pups treated with T4, but only the latter result was considered a “discovery” after controlling for FDR (q = 0.1544 and q = 0.0735, respectively) (Fig. 2b). In addition, RD, AD and MD values were not significantly changed by the treatment (Additional file 1). To account for a more global FA response to the T4 treatment, a single large ROI was manually delineated to the forebrain (an example ROI shown in Additional file 2) and all FA values were plotted as group histogram distributions (Fig. 3a). To quantitatively assess the apparent shift of FA distributions in the T4 group (Fig. 3a), individual subject histograms were fitted using Burr distribution (T4 group; Additional file 3, vehicle group; Additional file 4). The fit parameters (*c, k*) provided access to individual histogram distribution modes, means and medians. All three values (mode, mean and median) were significantly shifted towards higher FA-values in the T4 group, and medians are shown in Fig. 3b (*P < 0.05, Welch’s t-test).

### Development of the MBP ELISA

For protein quantification, ELISA has considerable advantages over conventional western blot and IHC techniques in terms of the speed, high throughput capacity and sensitivity. However, MBP protein expression analysis by ELISA in whole brain tissue lysates is challenging. Brain tissue is complex and contains “sticky” constituents that non-specifically bind MBP and interfere with traditional sandwich ELISA measurements due to high non-specific background signal [47]. MBP in diluted solutions binds to glass, plastic, certain brain proteins, as well as to red blood cells [48]. Additionally, the conventional ELISA methods do not have the sufficiently high sensitivity to enable detection of the low amounts of MBP in the developing rat brain. To this end, we have developed a novel MBP ELISA based on the direct method of detection (Sword Diagnostics, Chicago IL) [47] rather than on the tetramethylbenzidine-based sandwich approach, and coupled the horseradish peroxidase reaction with RRS reagents. This new method allowed to overcome the problem of non-specific binding and to increase the sensitivity of detection. The RRS-ELISA demonstrated higher sensitivity than the standard tetramethylbenzidine-based detection approach (Additional file 5). We also compared the direct ELISA to the more widely used sandwich ELISA and found the direct method to have higher sensitivity (LLOQ 1.563 ng/mL versus LLOQ = 25 ng/mL), leading to more reproducible MBP measurements. To perform the absolute quantification of MBP in neonatal rat brain lysate samples, MBP protein concentrations were fitted to a standard curve obtained with purified MBP standards (Additional file 6). Instead of using an anti-MBP capture antibody to coat the plate, as in the sandwich ELISA, we bound sample brain lysate to an uncoated polystyrene plate. Being aware that excess protein for antigen coating of polystyrene can reduce the sensitivity of the detection antibody, we ran a titration of different lysate dilutions (2.5–10 ng/mL) and determined the concentration of 10 ng/mL to be most optimal for the analysis.

A spike-and-recovery analysis was performed to confirm that our lysate sample matrix did not interfere with the standard assay diluent (Additional file 7). Our lysate samples also displayed ideal linearity when serially diluted. Most values from the spike, recovery, and linearity experiments fell within our acceptance range (70–120%), so values in that working range were determined with confidence.

### MBP analysis

**Fig. 4:**
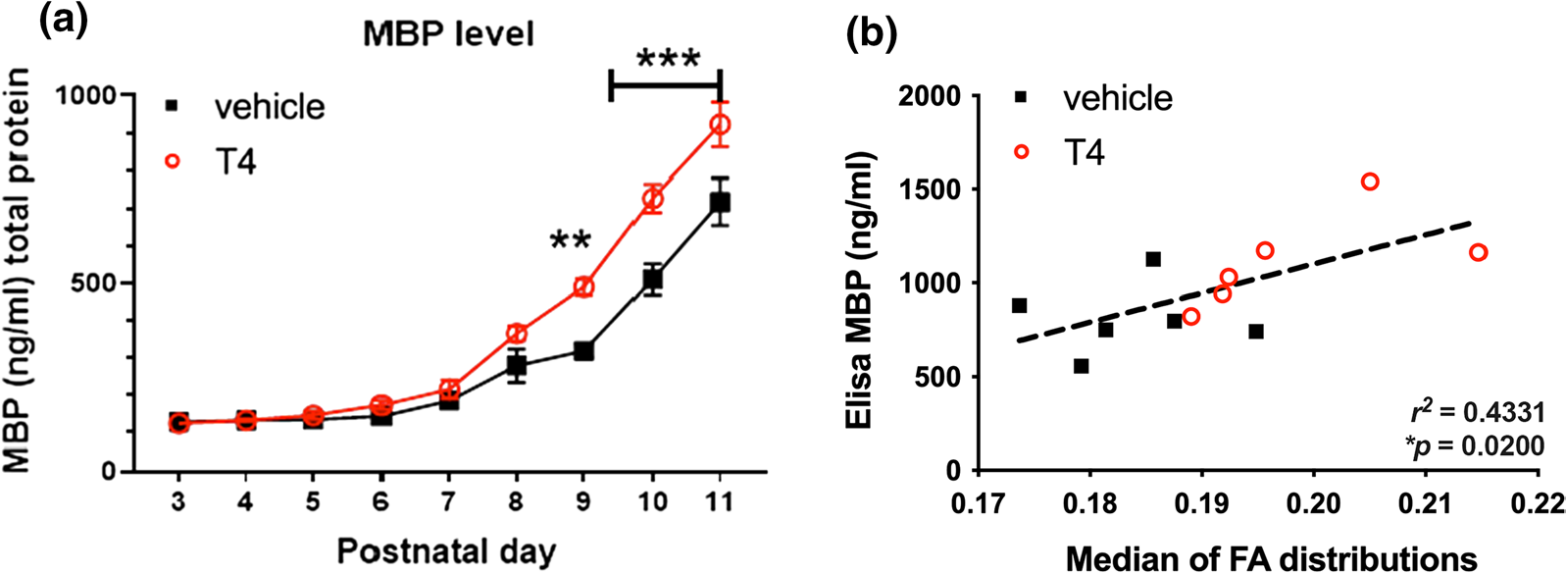
T4 facilitates myelination in the developing rat brain. The developmental increase in brain MBP levels in thyroxine (T4)- and vehicle-treated rats aged 3–11 days old was determined by Resonance Raman Spectroscopy ELISA (RRS-ELISA) in homogenized brain lysates prepared from brain hemispheres. **a** The relationships between brain lysate MBP level and age in the two treatment groups are illustrated. T4 exhibited age-dependent positive effect on MBP levels. Data are presented as the mean ± SEM (n = 5–7). Statistical significance of differences between MBP levels in the two treatment groups at the analyzed age points is indicated as follows: **P < 0.01; ***P < 0.001 (Tukey’s multiple comparisons test following two-way ANOVA). **b** A significant positive correlation between brain lysate MBP levels and median FA distribution value in rat pups that underwent DTI. Red empty circles and black filled squares denote T4-treated and vehicle-treated pups, respectively

MBP protein level in rat brain was assessed using the RRS-ELISA based on absolute MBP quantification with an MBP standard curve (Additional file 6). MBP protein levels in the brain of rat pups were significantly affected by (F_(8, 90)_ = 143.8, *P* < 0.0001), treatment (F_(1, 90)_ = 43.61, *P* < 0.0001) as well as by the treatment×age interaction (F_(8, 90)_ = 5.125, *P* < 0.0001). ANOVA with Tukey’s *post hoc* test showed that treatment of rat pups with T4 significantly increased MBP levels compared to that in the vehicle-treated groups after 6–9 days of administration (Fig. 4a). Furthermore, MBP levels in rat pups following nine daily administrations of T4 or vehicle correlated with DTI FA histogram medians calculated from Burr distribution fits (Pearson r^2^ = 0.4331, *P* = 0.0200) (Fig. 4b).

**Fig. 5:**
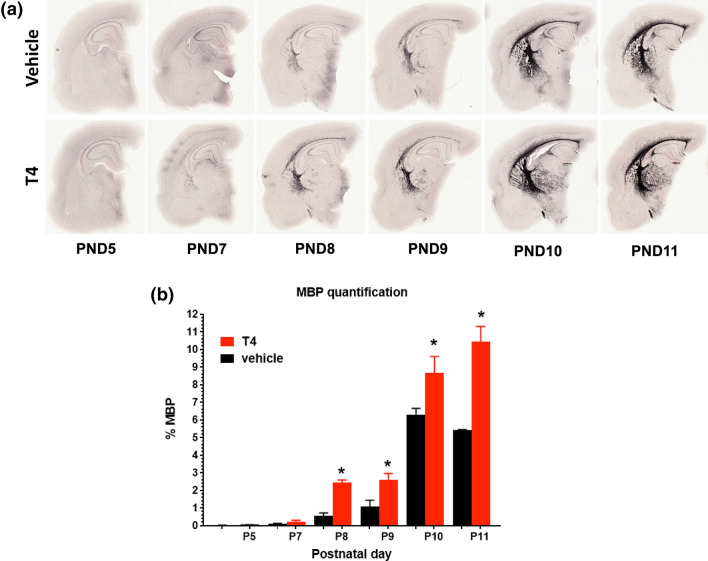
Analysis of immunohistochemical staining for MBP in coronal brain slices of vehicle- and T4-treated rat pups. **a** Representative examples of MBP protein staining after treatment with thyroxine (T4) or vehicle at PND5–11. **b** Quantification of the MBP signal optical density at PND5 and PND7–PND11. Six sagittal adjacent brain sections containing corpus callosum rostral to the hippocampus from at least two animals per group were used for this analysis. T4 treatment significantly increased MBP expression levels at PND 9 and 11. Data are presented as the mean ± SEM (n = 6). For the data quantification analysis, the regions of T4 vs. vehicle were compared to using the Student’s *t*-test and the correction for multiple comparisons was done by controlling for the false discovery rate (FDR; set at q = 0.1) by using the Benjamini–Krieger–Yekutieli procedure implemented in Prism 9. Data are presented as the mean ± SEM. Differences were considered statistically significant if adjusted *P* < 0.05. Asterisk (*) denotes P values of T4 compared to vehicle for each postnatal day,*P < 0.05 (Student’s *t*-test). Black and white bars denote T4-treated and vehicle-treated pups, respectively

IHC staining of rat coronal hemisphere sections for MBP indicated higher expression levels in T4-treated rat pups (Fig. 5a). Densitometric quantification of the images showed that T4 significantly increased MBP IHC signal at PND10 and 11 (*P < 0.05; ***P < 0.0001, ANOVA with Tukey’s *post hoc* test) ) (Fig. 5b). These results were in excellent correspondence with MBP protein level changes by RRS-ELISA (Fig. 4).

## Discussion

Preclinical assessment of novel promyelinating compounds in vivo is often a difficult and lengthy process because of the inherent limitations of animal models [49, 50]. For example, induced demyelination may require extended observation period (experimental autoimmune encephalitis [EAE] model) [51], prolonged treatment with the demyelinating agent to avoid spontaneous remyelination (cuprizone model) [52, 53], or technically demanding focal injections of toxins into the WM (lysolecithin and ethidium bromide models) [54, 55]. In this study, by using T4 as a known stimulator of myelination, we show that promyelinating effects may be detected within ten days in neonatal rats by using DTI measures. This was supported by the data from the simultaneous analysis of MBP protein levels in brain homogenates and by the quantification of IHC staining for MBP.

Thyroid hormones play an important role in myelination in normal development and in pathological conditions [31, 56]. Free T4 levels have been found to be proportional to the WM volume, although in older people, the relationship may be inverse [57]. In individuals with hypothyroidism, the total WM volume is usually decreased, which likely indicates impaired myelination [58, 59]. In agreement with data in humans, studies in preclinical models also showed that hypothyroidism leads to decreased WM volume as well as to deranged corpus callosum maturation and lower expression of myelin-related genes and proteins [60–62]. Neonatal hyperthyroidism in rats is associated with opposite changes [40, 60, 63], although prolonged hyperthyroidism may cause eventual apoptosis of oligodendrocytes [40]. Our present observations of the faster developmental increase in the expression of MBP in rat pups treated with T4 for a short period (Figs. 4a and 5a and b) are therefore in agreement with these published data.

The tight relationship between the level of thyroid hormones and myelination warranted exploration of T3 and T4 as myelination-promoting agents. Indeed, administration of T3 and T4 enhanced remyelination in the cuprizone and EAE models of demyelination by restoring MBP expression and activating oligodendrocyte precursors [33–35, 37–39, 64]. It has been proposed that thyroid hormones bind to thyroid hormone receptor β and upregulate expression of KLF9 (kruppel-like factor 9), which in turn regulates oligodendrocyte differentiation and myelin regeneration [65, 66]. Although, the clinical use of endogenous thyroid hormones to improve myelination may not be possible due to the adverse effects of hyperthyroidism, synthetic thyromimetics may be promising in this regard [67–69].

Given that several weeks or more may be required in the standard preclinical models to induce demyelination before the promyelinating candidates may be administered, we sought to develop a faster assay, in which myelination-enhancing action could be detected on a shorter time scale. We used DTI, an MRI technique sensitive to the integrity of WM, which is routinely used for the assessment of myelination disorders in clinical [70–73] and preclinical studies [74–76]. We chose to use T4 as a powerful promyelinating compound and in this respect, it should be noted that DTI has been used to detect myelination differences due to altered thyroid status both in humans and in animal models [38, 77]. In DTI, the strength and direction of water diffusivity in the brain are inferred from the values of radial, axial and mean diffusivities as well as from FA, which depends on the degree of coherence of water diffusion and is thought to be proportional to the extent of myelination of axonal fibers. In a simplified model for the extracellular water movement between the axons, the developmental myelination in rat pups could be expected to reduce the radial proportion of directional dependency, simultaneously increasing the FA values. Our analysis of DTI parameters in individual tracts showed higher FA values in the forceps minor of the corpus callosum and the internal capsule in T4-treated rat pups as compared to the values in vehicle-treated group at PND10, although the effect in the former region did not survive the correction for the false discovery rate (Fig. 2b).

Manual ROI analysis is based on the anatomical delineation of structures using available atlases and reference data, but it is also subjected to arbitrary thresholding of estimated diffusion indices, such as FA. Both the thresholding (limit perceived as high enough contrast in FA to account for WM structure) and manual delineation of structures are to some extent subjected to the off-tract-center partial voluming effect [78] that creates observer-dependent confounders even if blinded data analysis is conducted using similar criteria on all subjects and between the treatment groups. In case of the developing brain, this thresholding becomes even more arbitrary as FA of WM structures increases with brain maturation during early development [79, 80]. To avoid such confounders and to conduct an objective analysis of the global developmental myelination status, histogram analysis of FA-values was established from one single ROI spanning all relevant WM structures without thresholding. Analysis of group averages of normalized FA histogram distributions showed apparent shift of FA to higher values in the T4 group (Fig. 3a). Burr distribution fits to individual histograms and subsequent statistical analysis of individual histogram medians demonstrated that this shift towards higher FA values was significant (Fig. 3b). Although changes in FA values are often interpreted only in terms of myelin integrity, they may also be caused by other factors, e.g., orientation-dependent aspects of tissue microstructure or axonal pathology [81, 82]. Therefore, to confirm that the increase in FA following T4 treatment was indeed caused by myelin-related processes, we examined the relationship between FA distribution medians and MBP levels in hemisphere samples from the same animals and found a statistically significant correlation (Fig. 4b). Furthermore, DTI and RRS-ELISA data were also supported by the analysis of IHC images, which also showed that developmental increase in the amount of MBP proceeded faster in T4-treated pups than in vehicle-treated counterparts (Fig. 5a, b). Developmental changes in MBP expression have been used as an index of promyelination in studies employing fixed brain IHC [83] and *ex vivo* slice cultures [84]. Extending these approaches, we have introduced a comparative analysis based on a novel, rapid and ultra-sensitive MBP ELISA that can be easily set-up in most laboratories with a compatible plate reader.

## Conclusions

To the best of our knowledge, the neonatal model of myelination described in this paper is the only rapid in vivo model for testing promyelinating compounds to date. By using a combination of in vivo DTI and rapid in vitro biochemical and IHC staining approaches, we have provided thorough quantitative description of the promyelinating effect of T4. This approach will be useful not only for in vivo screening of test compounds for developmental hypomyelinating disorders, but also for pre-screening of test compounds prior to their tests in more expensive, longer-term animal disease models of demyelination (i.e. MS models), and thus, it may be a valuable drug discovery tool. Further experiments will be required to establish the sensitivity of this method for a wide range of known promyelinating agents and whether identified promyelinating compounds are also predictive of remyelination in disease models.

## Supplementary Information

**Additional file 1:** Radial diffusivity (a), axial diffusivity (b) and mean diffusivity (c) in 10 different areas of white matter determined using diffusion tensor imaging in 10-day-old rat pups following nine daily administrations of thyroxine (T4, 0.1 mg/kg, n = 6, red bars) or vehicle (water for injections, n = 6, black bars). Data are presented as the mean ± SEM. no significant discoveries after FDR correction for the false discovery rate at q set at 0.1. Annotations: fmi, forceps minor of corpus callosum; gcc, genu of corpus callosum; bcc, body of corpus callosum; scc, splenium of corpus callosum; fmj, forceps major of corpus callosum; ec, external capsule; aca, anterior part of anterior commissure; ic, internal capsule; opt, optic tract; cp, cerebellar peduncle.

**Additional file 2:** Representative region of interest (red color) overlaid with the fractional anisotropy (FA) map, from which the FA value-distributions were obtained for Fig. 3a.

**Additional file 3:** Individual forebrain fractional anisotropy (FA) distributions of all subjects in the thyroxine (T4) group. Red line is fitted Burr distribution overlaid with representative original raw data histogram. Burr distribution fit parameters (α, c, k) as well as mean (black line) and median (green line) values are shown in the panels.

**Additional file 4:** Individual forebrain fractional anisotropy (FA) distributions of all subjects in the vehicle group. Red line is fitted Burr distribution overlaid with representative original raw data histogram. Burr distribution fit parameters (α, c, k) as well as mean (black line) and median (green line) values are shown in the panels.

**Additional file 5:** Comparison of standard curves generated by using the resonance Raman spectroscopy/enzyme-linked immunosorbent assay (RRS-ELISA) and standard tetramethylbenzidine-based ELISA (TMB-ELISA) demonstrates the increased sensitivity of the former method. Values blank-subtracted and normalized (high of 100, 000) to compare on the same graph.

**Additional file 6:** An optimized MBP direct ELISA standard curve was achieved by determining an appropriate blocker, antibody concentrations, sample dilutions, sample incubation time and buffer system.

**Additional file 7:** Spike recovery and linearity analysis demonstrated that our lysate sample matrix did not interfere with the standard assay diluent. Given that high concentrations of MBP are not always known in the samples, testing was done by adding (spiking) known amounts of MBP into the samples.

## Data Availability

The datasets used and/or analyzed during the current study are available from the corresponding author on reasonable request.

## Abbreviations

AD: Axial diffusivity
ANOVA: Analysis of variance
DTI: Diffusion tensor imaging
EAE: Experimental autoimmune encephalitis
FA: Fractional anisotropy
FDR: False discovery rate
IHC: Immunohistochemical
MBP: Myelin basic protein
MD: Mean diffusivity
MRI: Magnetic resonance imaging
MS: Multiple sclerosis
PND: Postnatal day
RD: Radial diffusivity
ROI: Region of interest
RRS: Resonance Raman spectroscopy
T4: Thyroxine
WM: White matter
