# Supplementary figures and images for: An in vivo accelerated developmental myelination model for testing promyelinating therapeutics
Source: BMC Neurosci. 2022 May 25;23:30. doi: 10.1186/s12868-022-00714-y (PMC9134688; doi:10.1186/s12868-022-00714-y)

a)

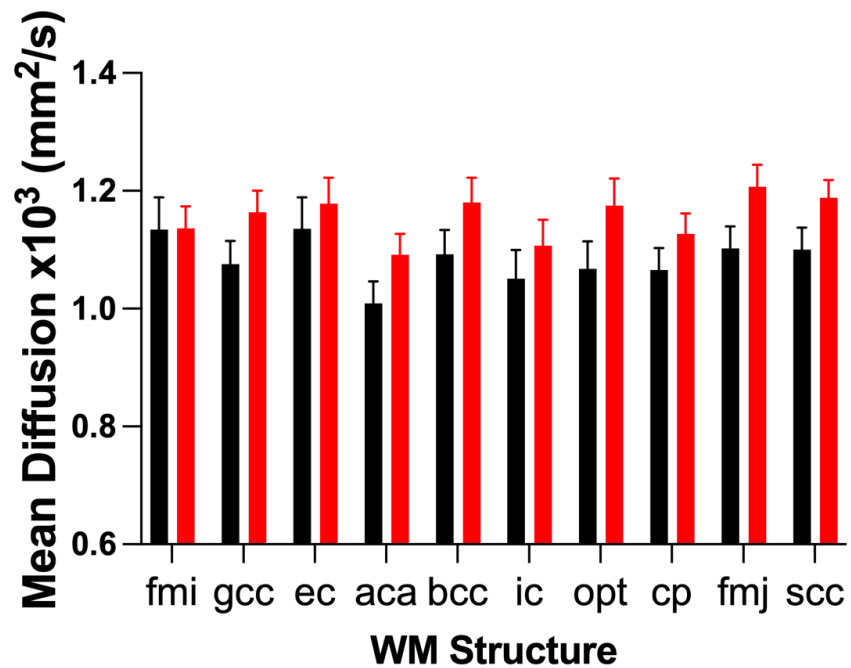

b)

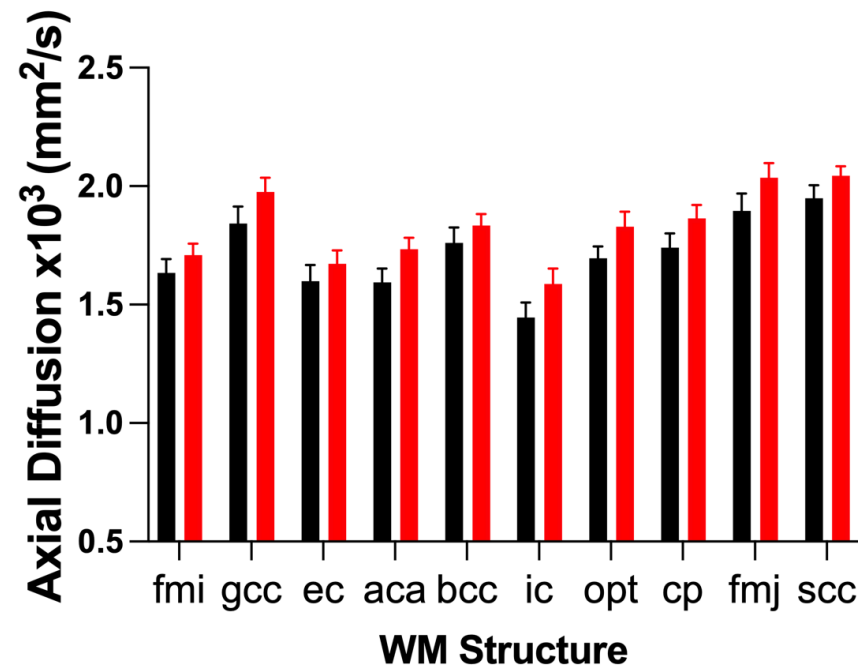

c)

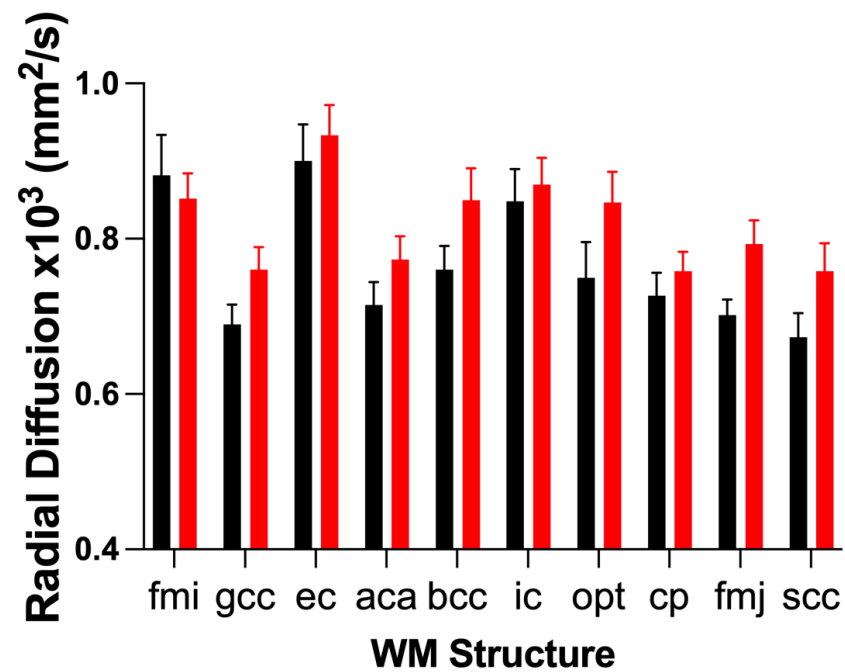

Supplement: Supplementary file 1 — Additional file 1: Radial diffusivity (a), axial diffusivity (b) and mean diffusivity (c) in 10 different areas of white matter determined using diffusion tensor imaging in 10-day-old rat pups following nine daily administrations of thyroxine (T4, 0.1 mg/kg, n = 6, red bars) or vehicle (water for injections, n = 6, black bars). Data are presented as the mean ± SEM. no significant discoveries after FDR correction for the false discovery rate at q set at 0.1. Annotations: fmi, forceps minor of corpus callosum; gcc, genu of corpus callosum; bcc, body of corpus callosum; scc, splenium of corpus callosum; fmj, forceps major of corpus callosum; ec, external capsule; aca, anterior part of anterior commissure; ic, internal capsule; opt, optic tract; cp, cerebellar peduncle. [file 12868_2022_714_MOESM1_ESM.pdf]

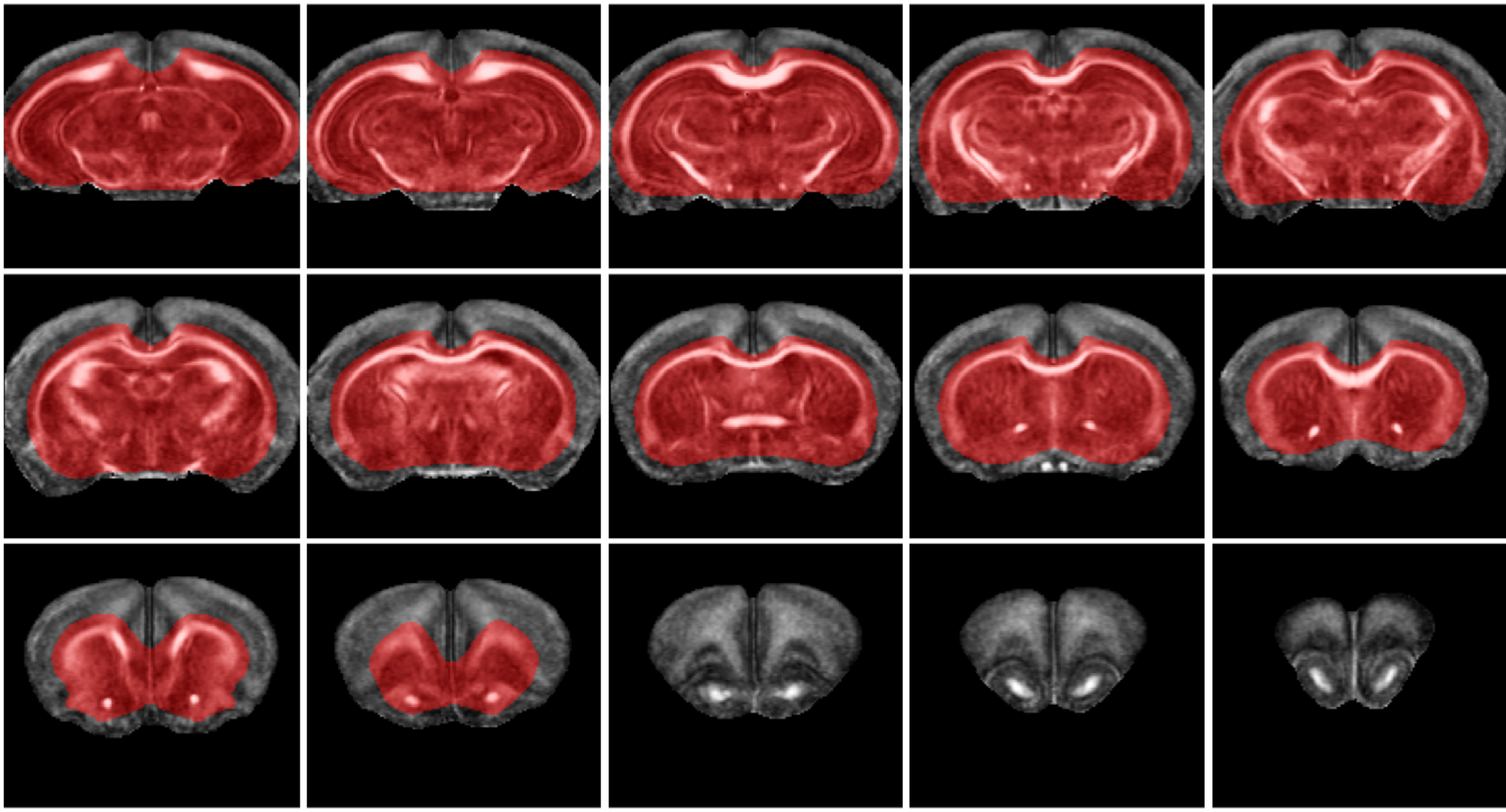

Supplement: Supplementary file 2 — Additional file 2: Representative region of interest (red color) overlaid with the fractional anisotropy (FA) map, from which the FA value-distributions were obtained for Fig. 3a. [file 12868_2022_714_MOESM2_ESM.pdf]

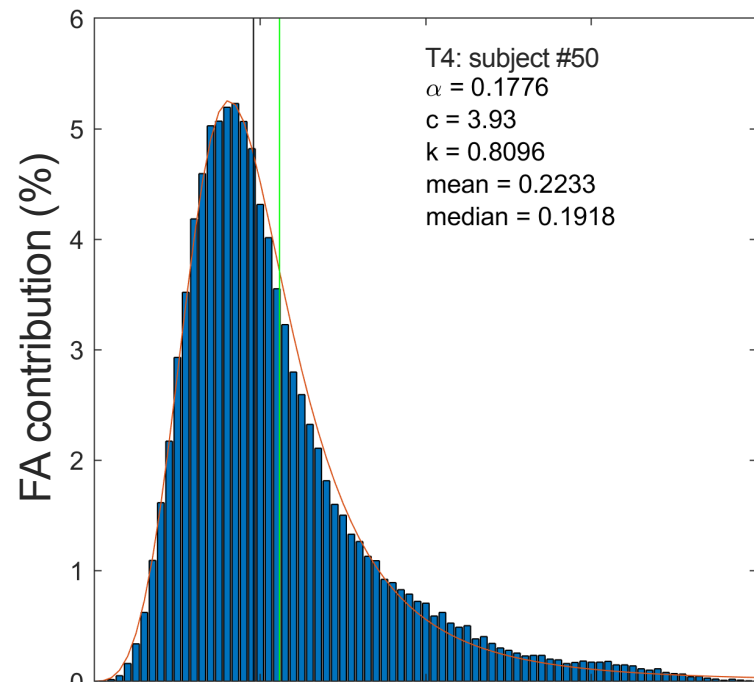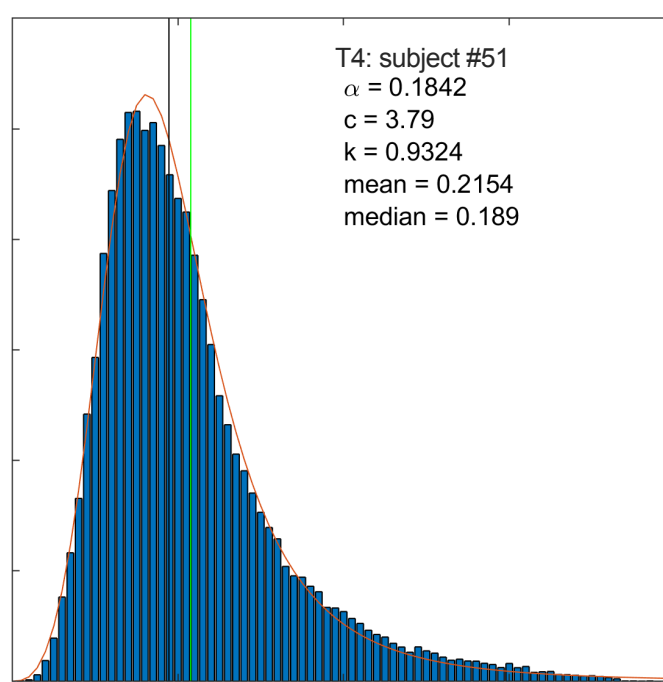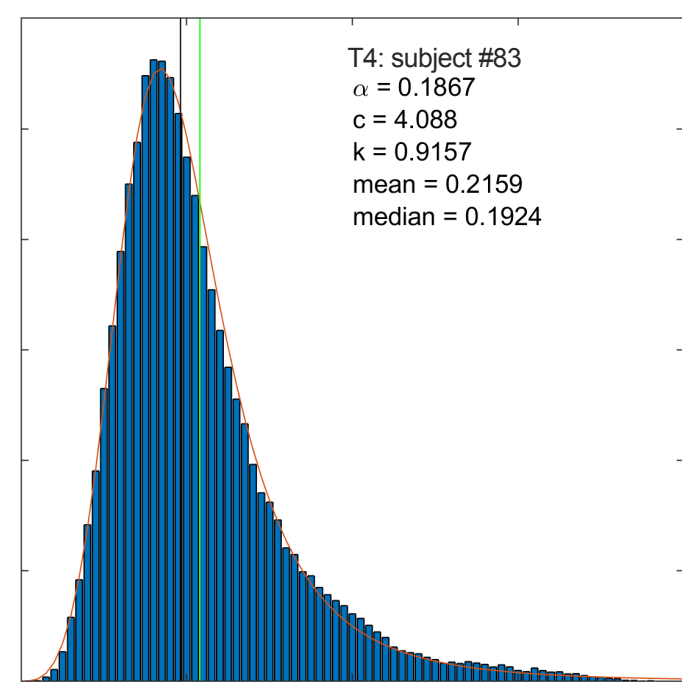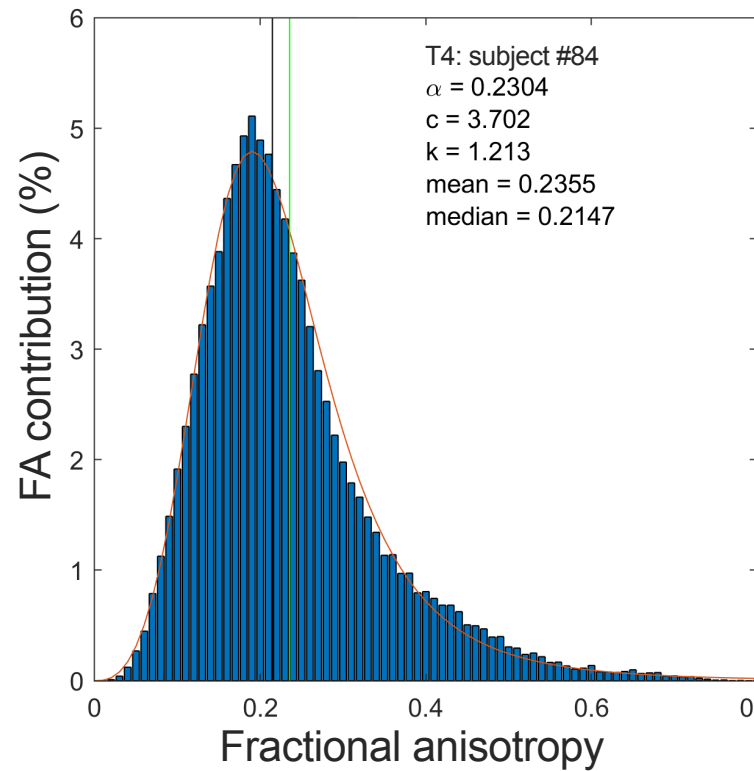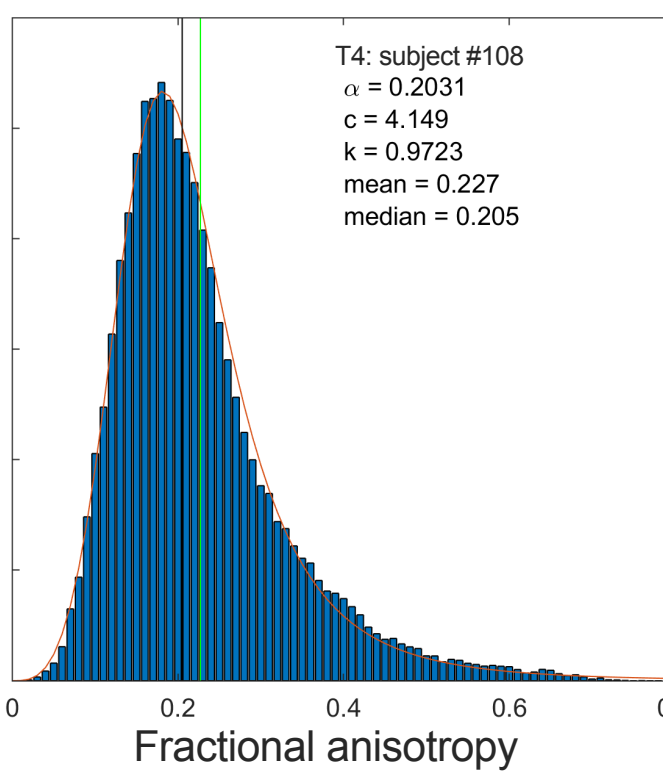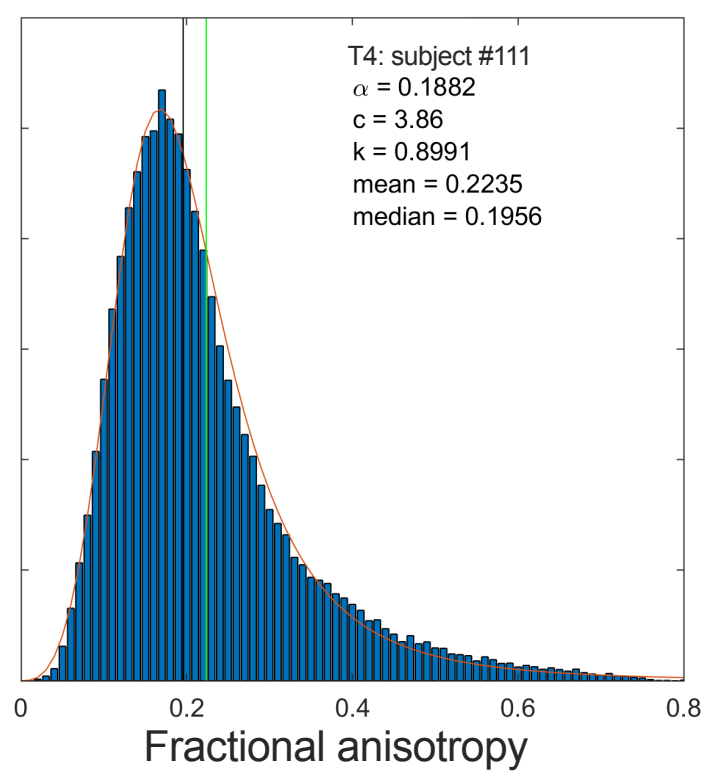

Supplement: Supplementary file 3 — Additional file 3: Individual forebrain fractional anisotropy (FA) distributions of all subjects in the thyroxine (T4) group. Red line is fitted Burr distribution overlaid with representative original raw data histogram. Burr distribution fit parameters (α, c, k) as well as mean (black line) and median (green line) values are shown in the panels. [file 12868_2022_714_MOESM3_ESM.pdf]

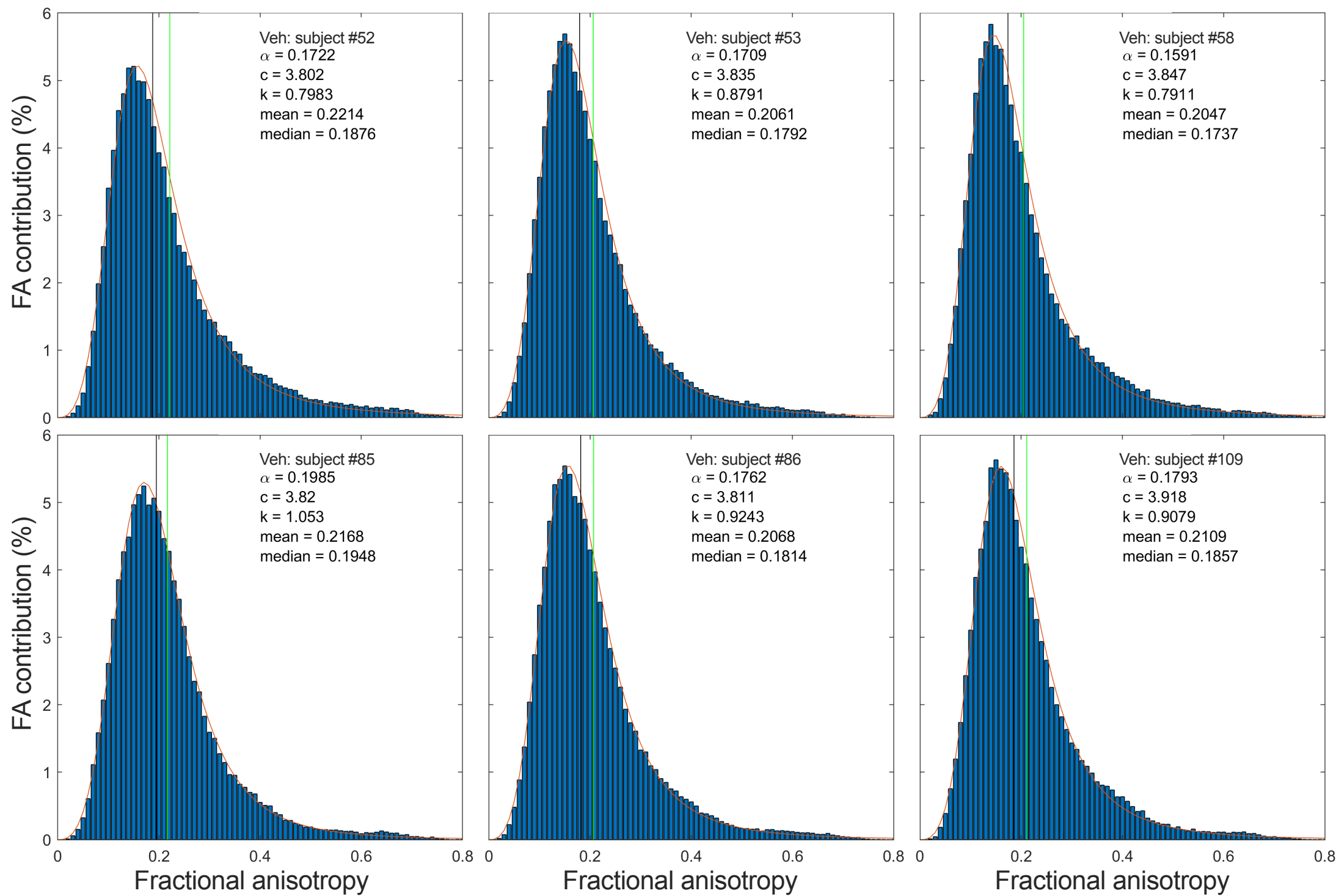

Supplement: Supplementary file 4 — Additional file 4: Individual forebrain fractional anisotropy (FA) distributions of all subjects in the vehicle group. Red line is fitted Burr distribution overlaid with representative original raw data histogram. Burr distribution fit parameters (α, c, k) as well as mean (black line) and median (green line) values are shown in the panels. [file 12868_2022_714_MOESM4_ESM.pdf]

**MBP Detection in Rat Brain Lysate**

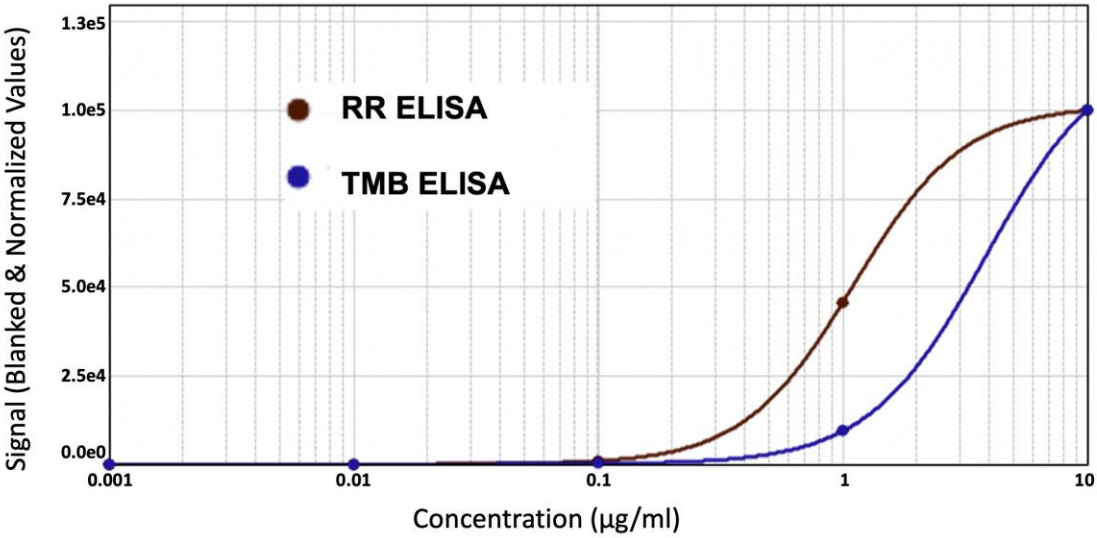

Supplement: Supplementary file 5 — Additional file 5: Comparison of standard curves generated by using the resonance Raman spectroscopy/enzyme-linked immunosorbent assay (RRS-ELISA) and standard tetramethylbenzidine-based ELISA (TMB-ELISA) demonstrates the increased sensitivity of the former method. Values blank-subtracted and normalized (high of 100, 000) to compare on the same graph. [file 12868_2022_714_MOESM5_ESM.pdf]
