# Supplementary material for: An in vivo accelerated developmental myelination model for testing promyelinating therapeutics
Source: BMC Neurosci. 2022 May 25;23:30. doi: 10.1186/s12868-022-00714-y (PMC9134688; doi:10.1186/s12868-022-00714-y)

Optimized MBP Direct ELISA Standard Curve

Sample	Concentration ng/mL	Values	Mean Value	Back-Calc	Mean Back-Calc	Accuracy	N	Std. Dev.	CV%
01	0.000	33031<br>33432<br>33328	33263.7	Range?<br>Range?<br>Range?	Range?	Range?	3	208.10	Range?
02	0.391	Masked<br>34781<br>34348	34564.5	Masked<br>0.578<br>0.421	0.499	127.63	2	306.18	22.25
03	0.781	37102<br>34540<br>35515	35719.0	1.241<br>0.493<br>0.808	0.847	108.51	3	1293.13	44.32
04	1.563	38848<br>38207<br>37972	38342.3	1.674<br>1.518<br>1.460	1.551	99.22	3	453.41	7.14
05	3.125	44164<br>44428<br>44555	44382.3	2.948<br>3.012<br>3.044	3.001	96.04	3	199.46	1.63
06	6.250	60039<br>55069<br>53837	56315.0	7.864<br>6.023<br>5.620	6.502	104.04	3	3283.38	18.40
07	12.500	69746<br>68899<br>67723	68789.3	12.912<br>12.368<br>11.651	12.310	98.48	3	1015.95	5.14
08	25.000	82895<br>82137<br>82016	82349.3	25.883<br>24.812<br>24.646	25.114	100.46	3	476.42	2.67

Mean + 2SD of BKG = 33680 LOD = 0.391 LLOQ = 1.563

Re-optimized blocker, antibody concentrations, sample dilutions, sample incubation time and buffer system

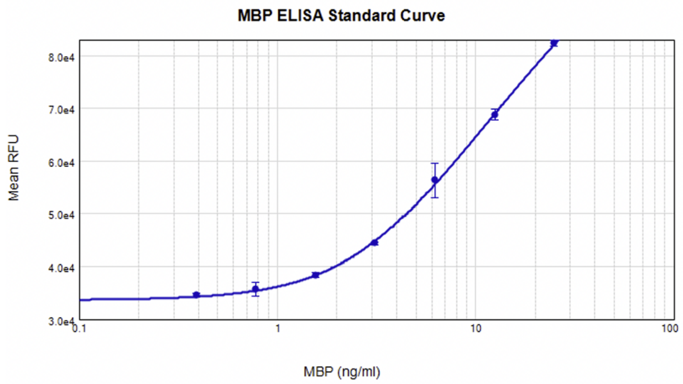

Supplement: Supplementary file 6 — Additional file 6: An optimized MBP direct ELISA standard curve was achieved by determining an appropriate blocker, antibody concentrations, sample dilutions, sample incubation time and buffer system. [file 12868_2022_714_MOESM6_ESM.pdf]
