# Supplementary material for: An in vivo accelerated developmental myelination model for testing promyelinating therapeutics
Source: BMC Neurosci. 2022 May 25;23:30. doi: 10.1186/s12868-022-00714-y (PMC9134688; doi:10.1186/s12868-022-00714-y)
Supplement: Supplementary file 7 — Additional file 7: Spike recovery and linearity analysis demonstrated that our lysate sample matrix did not interfere with the standard assay diluent. Given that high concentrations of MBP are not always known in the samples, testing was done by adding (spiking) known amounts of MBP into the samples. [file 12868_2022_714_MOESM7_ESM.pdf]

Spike Recovery		
Spiked MBP ng/L (n=3)	%CV	% Recovery
0.195	7.566	71.85
0.391	5.199	53.61
0.781	10.490	59.26
1.563	11.091	91.38
3.125	0.329	76.86
6.250	24.320	101.40
12.500	18.489	119.33
25.000	2.501	102.53

Unspiked Dilutional Linearity			
Sample concentration µg/ml (n=3)	Dilution	%CV	Linearity
5	1:1	15.943	100
2.5	1:2	0.208	126.25
1.25	1:4	54.186	92.22
0.625	1:8	15.817	202.07
